# A 5-year examination of CAPABLE implementation using RE-AIM and CFIR frameworks

**DOI:** 10.3389/fpubh.2025.1569320

**Published:** 2025-06-26

**Authors:** Deborah L. Paone, Jeanne W. Schuller, Matthew Lee Smith, Laura N. Gitlin, Sarah L. Szanton

**Affiliations:** ^1^Paone & Associates, LLC, Minneapolis, MN, United States; ^2^Johns Hopkins School of Nursing and the Bloomberg School of Public Health, Baltimore, MD, United States; ^3^School of Public Health, Texas A&M University, College Station, TX, United States; ^4^Center for Community Health and Aging, Texas A&M University, College Station, TX, United States; ^5^Drexel College of Nursing and Health Professions, Philadelphia, PA, United States

**Keywords:** implementation, implementation effectiveness, RE-AIM, CFIR, sustainability, CAPABLE, evidence-based, program funding

## Abstract

**Background:**

Examining the experience of organizations implementing evidence-based programs can help future programs address barriers to effective implementation, sustainment, and scaling. CAPABLE is an evidence-based 4-to-6-month program that improves daily function of older adults and modifies their home environments in modest ways to support their goal attainment. Through a guided process, utilizing an occupational therapist, nurse, and handy worker, the older adult sets goals and a personal action plan. In this study, we examined factors that advanced or impeded implementation and sustainability of CAPABLE. The researchers are embedded in the CAPABLE National Center and Johns Hopkins and provide ongoing technical support in implementation and dissemination of CAPABLE throughout the U.S., Canada, and other countries.

**Methods:**

We chose the RE-AIM and CFIR frameworks based on their robust use in the U.S. for examining implementation of older adult health promotion and prevention programs. We examined the implementation and sustainment experience of 65 organizations adopting CAPABLE across 5 years (2019–2024). Data sources included licensure records, an annual survey, and additional notes collected *ad hoc*. We identified key components to implement CAPABLE and used self-reported data from the lead program administrator at each organization who replied to the annual survey. These key informants responded to the level of ease or difficulty of these key components required for implementation. They responded each year that their organizations provided CAPABLE. CAPABLE licensure records indicated when the organization began/terminated their service. Notes from monthly office hours calls provided additional contextual information. We performed qualitative thematic and descriptive analysis on the notes. We also reviewed published studies on CAPABLE’s outcomes. The unit of analysis was the organization.

**Results:**

The following factors were consistently reported by these administrators as supporting ease of implementation: getting leadership support, accessing technical assistance, and maintaining fidelity to the program. Conversely, common challenges reported included difficulty with recruitment, hiring/finding the required personnel, and sustainability funding. Internal factors supporting readiness and adoption were perceived value of the program and program manager knowledge and commitment. External factors reported that supported adoption was initial funding to start a pilot, and alignment with “aging in community” strategic goals.

**Implication:**

This examination revealed positive and impeding forces for implementation and sustainment and identified where additional support was needed. Findings are guiding the development of this additional technical support by the CAPABLE National Center. In addition, efforts are underway to improve funding and policy to support CAPABLE to improve sustainment, scaling, and dissemination. This study also provides a use case for employing the RE-AIM and CFIR frameworks together to track ongoing implementation. This helps address a gap in the literature concerning practical ways to monitor, evaluate, and report on ongoing implementation of evidence-based programs.

## Introduction

Understanding the implementation and dissemination experience of organizations as they implement and sustain evidence-based programs can help future programs address barriers to sustainment and scaling. Organizations implementing evidence-based programs are motivated to effectively launch, operate, and sustain these programs, but they must overcome internal and external hurdles to do so (1). Studies of implementation case examples have found the interplay between program features, organizational characteristics, and external environments impact success (2–9).

Community Aging in Place—Advancing Better Living for Elders (CAPABLE) is a structured, 4- to 6-month home visit program to improve physical function among older adults by addressing individual capacity and modifying the home environment (10, 11). It uses an interprofessional team composed of an occupational therapist (OT), registered nurse (RN), and handy worker in an iterative series of home visits to assist the older person to attain self-determined goals and build capacity for self-care, deploying techniques of motivational interviewing and client-directed action.

Since 2009, CAPABLE has been tested in randomized control trials and demonstrated improvement in activities of daily living (ADL) such as bathing, dressing, and eating (8 ADLs examined) and instrumental activities of daily living (IADL) such as paying bills, grocery shopping (8 IADLs examined), functional status, and depression (12). Studies about the effect of CAPABLE on healthcare utilization and costs showed cost savings (13–15). Examining Medicare expenditures for CAPABLE participants relative to comparators in a study sample of 5,861 beneficiaries, CAPABLE had lower expenditures driven by reductions in inpatient and outpatient expenditures. Expenditures were lower by $2,765 USD per participant per quarter for eight consecutive quarters, which totaled $22,120 USD per participant over 2 years (14). Examining Medicaid expenditures, these were lower an average of $867 USD per participant per month due to lower utilization and spending in all healthcare services except home health (15). For people dually eligible for Medicare and Medicaid, savings would be approximately $30,000 in 2017 US dollars ($2,765 × 8 quarters plus $867 × 24 months).

While the efficacy and effectiveness of CAPABLE is widely understood, less is reported about the important aspects of program implementation that have facilitated its diffusion into communities across the United States and Canada. In this context, the purposes of this examination were to (1) identify factors that are important (as facilitators or hurdles) to organizations when implementing and sustaining CAPABLE; (2) assess the utility of using two frameworks to study implementation of CAPABLE by a range of organizations at various stages of implementation progress; and (3) provide practical recommendations to advance the dissemination of CAPABLE, which can be adopted/modeled by other evidence-based programs.

### Framework selection

We chose the Reach-Effectiveness-Adoption-Implementation-Maintenance (RE-AIM) and the Consolidated Framework for Implementation Research (CFIR) frameworks to guide our examination, given experience by the lead researcher with these frameworks and extensive use in the field, especially in examining implementation and dissemination of health promotion programs for older adults (17–22, 25–27, 41, 42).

The five domains of the RE-AIM framework (i.e., Reach, Effectiveness, Adoption, Implementation, and Maintenance) were important to our examination to provide a macro-view of the 5-year implementation experience of CAPABLE. RE-AIM is used for planning and evaluation and can help determine impact of an evidence-based program (16–22). The Practical, Robust Implementation and Sustainability Model (PRISM) is an evolution of RE-AIM designed to improve translation of research into community practice (23) which we examined but did not use. Researchers in implementation science have noted the importance of continuing to apply RE-AIM iteratively as part of ongoing evaluation in a measurement and improvement cycle (17, 20, 21, 24, 44).

We chose CFIR to examine specific factors that are important at an organizational level. CFIR provides a structured way to examine specific constructs and context as organizations implement a given program. The researcher selects constructs based on understanding program components and internal and external aspects of readiness (25). Since the study team had been involved in shepherding the CAPABLE program, working with organizations directly, we were able to use this knowledge to confidentially select internal and external factors, consistent with the guidelines around the use of CFIR. In this way, CFIR provided us with a structured way to conduct a more granular focus at the organizational level. As stated by Damschroder, capturing setting-level barriers and facilitators to predict or explain antecedents and implementation outcomes is the appropriate use of CFIR (26).

Both RE-AIM and CFIR have robust research communities and Internet-based applications with open-access tools and resources. Each framework has been used for more than two decades, and there is a strong body of literature available^1^^,^^2^. These two frameworks have been used effectively together (27). An addendum to CFIR provides conceptual distinctions and connects RE-AIM and CFIR to guide analysis on outcomes (26).

Our approach follows the recommendation to move beyond the conceptual or theoretical use of a framework to where it is incorporated and operationalized within the implementation effort (28). The 10 recommendations offered by these researchers stress the use of the frameworks in “real-world” implementation projects. Therefore, we attempted to follow their 10 recommendations in this examination which included selecting suitable frameworks, engaging the stakeholder (the organization) directly, having embedded researchers, defining key issues and implementation phases, identifying influences on the organization to inform a logic model, determining the methods for examination that were practical and would continue beyond this study in order to serve as an ongoing monitor, identifying key barriers and enablers that influence the outcome of effective implementation and sustainment, specifying the outcomes and monitoring progression, and using the framework at a micro-level to tailor support. The final step of reporting the implementation effort is this study and article (28).

## Methods

### Implementation stages

**Figure 1:**
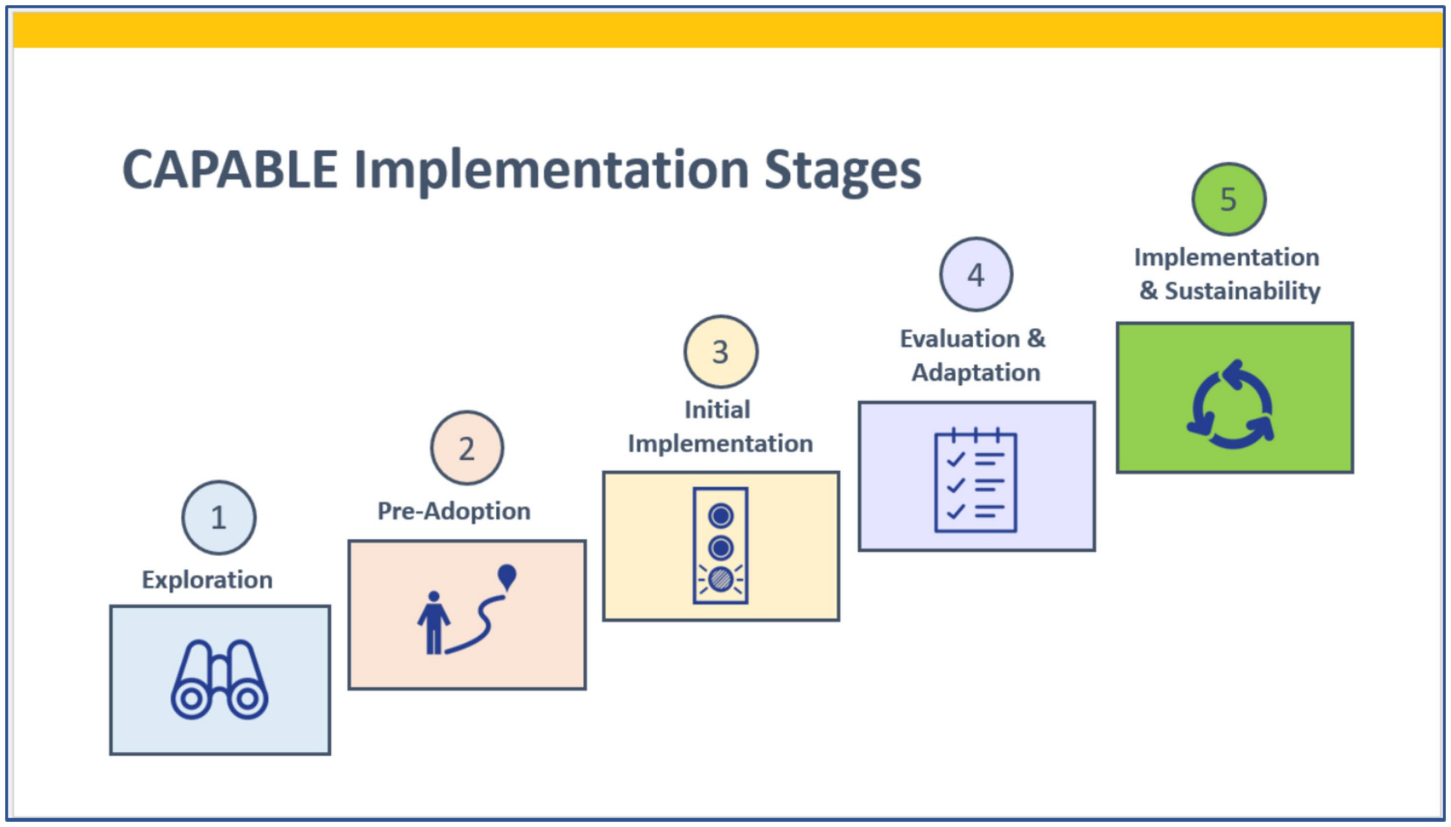
Implementation journey map. Adapted from the National Implementation Research Network (43, 45, 46).

On an ongoing basis, since 2019, the CAPABLE team has used a five-stage implementation journey map to support each organization as it moves from exploring CAPABLE to sustaining it (Figure 1) (45, 46). During Exploration of CAPABLE (*Stage 1*), organizational leaders review evidence on expected outcomes, the program protocol, and operational considerations. The Pre-Adoption stage (*Stage 2*) involves multiple contacts with the CAPABLE technical team to discuss “what it takes to launch CAPABLE.” In *Stage 3*, Initial Implementation, a program manager is assigned by the organization, and this person is tasked with launching CAPABLE—within a defined timeframe, budget, and scope. Hallmarks of the Evaluation and Adaptation stage (*Stage 4*) are reviews of the results from the initial implementation of the program. A critical component of Stage 4 is to secure ongoing funding. Leadership from the organization examine the resources and level of effort expended from their actual implementation experience (costs, labor inputs, internal processes required) and compare this to the outcomes attained (service volume, pre/post-health outcomes, receptivity of the program by target groups). Finally, *Stage 5* (Sustainability) is reached when the organization has completed their initial implementation stage and made the determination to continue. In this examination, we focus on organizations at Stages 3 through 5.

### Data sources

We reviewed all (*N* = 65) CAPABLE licensure records, which indicate when the organization signed their agreement to allow adoption of CAPABLE under the conditions specified by Johns Hopkins University. Licensure records include the organization address, program administrator name, date when the license contract was signed, payment received, and termination date (when the license expires if it is not renewed). Most licensure agreements were for a term from 2 to 4 years.

We reviewed all (*N* = 101) responses to our annual CAPABLE Implementation Check-in & Fidelity e-survey which is required for all licensed sites. The annual CAPABLE Implementation Check-in & Fidelity e-survey is conducted at the end of each year and responses are given at the organization level. Data collected include the number of participants served in that year, the organization’s intent to continue or end the CAPABLE service, staffing changes, cost per participant, funding sources, self-reported ease or difficulty of key implementation steps at the organizational level, self-reported organizational readiness to implement CAPABLE (in retrospect), and attestation that the organization has followed the program protocol with fidelity. The CAPABLE program administrator is the respondent for the organization and is the key informant for this examination. This provides the CAPABLE National Center program office with a core set of information on each site annually.

To create the survey items, we used our knowledge about each of the organizations that had adopted CAPABLE up to the point of this study. The lead author is the national program office key contact for technical support on implementation and, with other team members, had extensive first-hand knowledge of each of the 65 organizations. We identified 12 key implementation components for CAPABLE that were common across all implementation sites. These 12 components were probed via the CAPABLE annual e-survey with forced choice response using an adjectival Likert scale, from *Very Easy/Easy* to *Difficult/Very Difficult* (Table 1). Program administrators are the respondent, and they determine the level of ease or difficulty that they report. The administrators received the survey in December of each year and responded by the end of January to describe implementation progress for the previous calendar year. The lead author exported the Excel spreadsheet generated from the e-survey platform and aggregated the data calculating frequency and percentage for each level of the scale, by item.

**Table 1:** Implementation components.

Description of implementation components
1. Managing the program and overseeing the implementation of CAPABLE
2. Getting senior leadership support
3. Finding staff (OT, RN, handy worker)
4. Finding partners (e.g., healthcare and housing repair)
5. Setting up the workflows/processes
6. Recruiting participants & getting eligible referrals
7. Securing legal, insurance, business, or HIPAA agreements
8. Data & Evaluation—gathering and entering data, aggregating and evaluating the program (two items)
9. Participants completing the full program (at least 8 visits)
10. Developing a sustainable financial/funding model
11. Ensuring fidelity; monitoring how well the CAPABLE model is followed
12. Obtaining implementation assistance and guidance from the team

For additional contextual information, we reviewed notes from the monthly office hours with program administrators, which began in October 2019. There were approximately 60 calls held by June 2024 with brief notes available on most of them. The monthly call is akin to an “open office hour” for a drop-in discussion and open-ended, non-scripted conversation. The purpose of the calls is to foster shared learning between site program administrators and help ensure consistency across geographic settings and over time in how the program operates. All program administrators participated in at least one call during the term of their license agreement. The lead author has facilitated these monthly calls since their inception.

These monthly “office hours” calls have no specific agenda and thus represent an organic capture of questions, issues, and strategies. The calls offer a real-world (not surveyor or researcher-driven) perspective of implementation. As such, the notes are not structured, do not include direct quotes or identify the speaker, and are very brief. We believe these calls offer an important opportunity to the national program office to have a deeper understanding of operational issues, strategies, and concerns in real time. Such calls offer a convenience sample of organizational feedback about CAPABLE implementation strategies, questions, barriers, and facilitators. The notes represent a non-structured qualitative dataset.

In reviewing notes, we identified themes that were consistent with the implementation components and factors queried in the annual survey. We noted the commenter’s perspective on whether the component or issue was a positive or negative factor. This was straightforward as the commenter would indicate a challenging or an emerging enabling issue in the beginning of making their comment. We took at face value the determination of that person who is running the CAPABLE program as to whether a factor or issue they were describing was having a negative or a positive effect on their implementation progress. We did not adjudicate their opinions. We categorized these unstructured, *ad hoc* comments into the implementation components and constructs probed in the annual survey and used the contextual information to indicate the direction and strength of the factor.

No Institutional Review Board was required as these program data are collected as part of the licensure agreement between the organization and the CAPABLE National Center.

**Figure 2:**
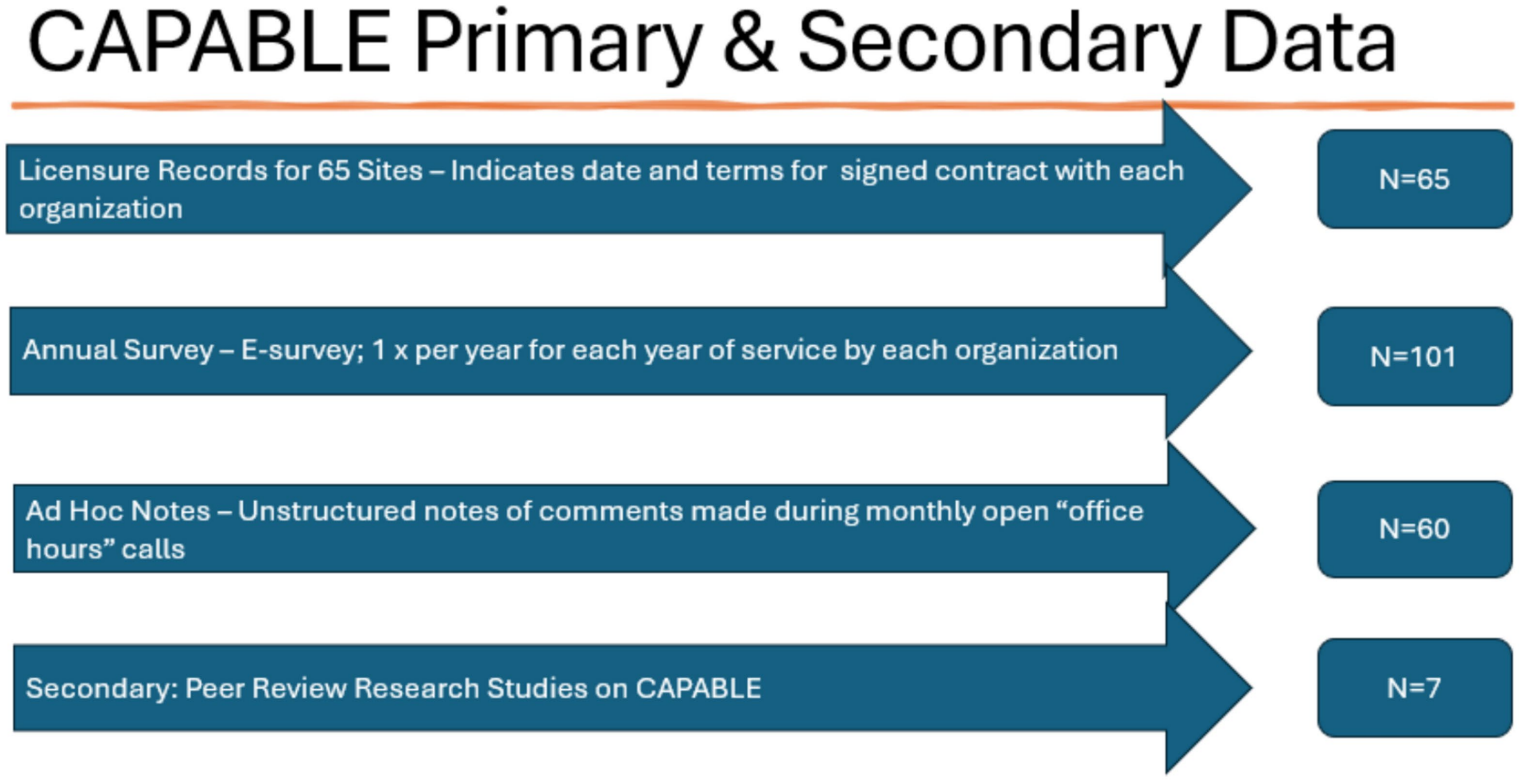
CAPABLE primary and secondary data.

The primary and secondary data are summarized in Figure 2.

To define effectiveness for this evidence-based program (what program outcomes are defined and proven for CAPABLE), we reviewed peer-reviewed published studies about CAPABLE program results (11–15, 29).

### Indicators

We selected seven indicators for examining macro-level results using the RE-AIM framework across the five domains of Reach, Effectiveness, Adoption, Implementation, and Maintenance (Table 2). We selected 13 constructs across the five areas within CFIR to examine internal characteristics, external environment, and other contextual factors that influenced CAPABLE implementation (Table 3). Construct selection was based on studies of implementation of evidence-based health promotion and disability prevention programs for older adults and our knowledge over 5 years working with the organizations implementing CAPABLE (5–7, 9, 16).

**Table 2:** RE-AIM indicators.

RE-AIM domains	CAPABLE indicators (overall results)	Data sources
Reach	# CAPABLE implementation sites (cumulative) # Participants served (cumulative)	Licensure records Annual Survey
Effectiveness	Outcome evaluation results (where available) on ADL, IADL, depression change pre to post for participants completing CAPABLE	Published research
Adoption	# Organizations starting up the program each year	Licensure records
Implementation	Self-reported implementation experience at the organizational level by program managers–Likert scale on ease/difficulty, and strength/direction of key components in terms of facilitating/impeding implementation Self-reported fidelity to CAPABLE protocol by organization	Annual Survey and monthly call notes
Maintenance	# Organizations sustaining operations beyond initial implementation	Licensure records and Annual Survey

**Table 3:** CFIR constructs.

See CFIR website: https://cfirguide.org/	Organizational constructs
Intervention characteristics	1. Evidence strength and quality
	2. Complexity
	3. Cost
Outer setting	4. External policy and incentives
Inner setting	5. Structural characteristics
	6. Readiness for implementation
	7. Leadership engagement
	8. Available resources
	9. Access to knowledge and information
Characteristics of individuals	10. Knowledge and beliefs about the intervention
Process	11. Planning
	12. Formally appointed internal implementation leaders
	13. Executing

The annual e-survey contained items on the 12 implementation components and 13 CFIR constructs listed in Tables 1, 3. This allowed us to systematically collect consistent data on every licensed CAPABLE site at least annually and round out our understanding with the Program Administrator monthly “office hours” virtual meetings. Every site participated in at least one call, and all except three organizations participated in the annual survey at least once. Since the CAPABLE service for most of these organizations extended from 2 to 4 years, some administrators responded multiple times. There is utility in continuing to ask administrators annually about their implementation experience as ease or difficulty with different components varies from year to year.

Respondents rating for each of the 12 implementation components were tallied, and the percentage distribution across the Likert scale from *Very Easy* to *Very Difficult* was calculated. As mentioned, it is the perception of the program administrator of that CAPABLE site who determined the ease or difficulty of that implementation component. In other words, we took at face value the determination of that person who is running the CAPABLE program as to whether a factor or issue had a negative or a positive effect on their implementation in the survey years and how strong of an effect was experienced. We did not adjudicate their opinions. There are no pre-defined cut point thresholds for each adjective level in the scale.

We examined self-reported readiness from high to low for each of the 13 constructs for each organization as indicated in the annual survey. We coupled the respondents’ answers given in open-ended optional response boxes for items in the annual survey with qualitative information gathered through monthly “office hours.” This helped us to determine the strength and direction of the response on these constructs for their organization from “Strongly Positive” to “Strongly Negative.” Given that we were able to observe and interact with these administrators and teams from these organizations over multiple years, we had the opportunity to discuss organizational challenges and strengths several times one-on-one to confirm our categorization around these constructs. The items that were more frequently reported as “Very Difficult to Somewhat Difficult” were considered “Negative,” and the items that were more frequently reported as “Very Easy to Somewhat Easy” were considered “Positive”.

### Analysis

Our analysis for each of the domains in the RE-AIM framework involved examining the indicators shown in Table 2, with quantitative results (frequencies and percentages) and qualitative results (categorized comments and open-ended responses). The indicators for Reach were the cumulative number of implementing organizations and the number of participants they served collectively. The indicators for Effectiveness were the reported outcomes observed in the studies and by each organization in terms of aggregate change in the prescribed pre/post-measures (i.e., the overall aggregate change response observed by the organization). The site reported that their CAPABLE participants showed one of the following: “improvement,” “no change,” or “decline.” The indicator of Adoption was the number of organizations signing a license agreement and starting up each year. The indicators of Implementation were the self-reported implementation experience by the program administrator for each of 12 implementation components and their attestation of fidelity to the protocol. The indicator of Maintenance was the number of organizations sustaining their operations beyond the initial implementation period, which was usually 2 to 4 years.

The analytical strategy for all of the implementation factors was frequency counts and percentages. We examined each year’s survey results and then looked at the trends or patterns year over year. The analytical strategy for the 13 constructs probed was also frequency counts of self-reported organizational readiness as indicated by the program administrator. The analytical strategy for the contextual qualitative information provided *ad hoc* in the calls was categorization of brief notes by common themes categorized by the 12 implementation components and 13 constructs. For the survey and the ad hoc comments in the monthly calls, we took at face value the positive or negative direction of the program administrators’ comments as they indicated their experience on the implementation components or constructs.

We examined the perspective of each organizational respondent one at a time and then aggregated results across all organizations. The data sources to determine the strength and direction in the aggregate were as follows: (1) the annual survey—where the person indicated their experience with the component using the Likert adjectival scale, (2) the annual survey—where the person sometimes provided additional open-ended responses about the organization’s experience, and (3) the monthly “office hours” calls, which the lead author facilitated and where the emphasis on a positive or negative component or construct was clear based on tone and language of the commenter. These data sources allowed us to count frequencies around how often an issue was raised by the 65 organizations (over time) and the direction of the comment. In this way, the strength and direction of the component or construct was determined by the frequency of it being raised and how emphatic the commenter was in terms of it being a barrier or facilitator. Although we did not have transcribed notes, some of the factors were so often raised that we could refer to our casual notes and memory of the conversations. A great advantage was having an embedded researcher facilitate all 60 calls. Directly providing technical assistance and conducting the calls over the 5 years studied provided an in-depth understanding of each organization and its strategy, challenges, and administrator’s perspective in real time and over time.

## Results

### Adoption

Over the 5 years studied by June 2024, 65 organizations had secured a license to implement CAPABLE, with 63 (96%) of these organizations progressing to initial implementation. Two organizations were licensed but never implemented the service; therefore, there was no administrator to answer the annual survey and no data to review for these organizations.

From 4 to 11 organizations became licensed each year in the 5 years studied. We do not have data on how many organizations may have initially considered CAPABLE but did not contact the CAPABLE National Center to indicate their interest in adopting the program.

At the end of each year, the CAPABLE annual survey was e-mailed to the program administrators within all licensed sites. This means that most of the organizations/program administrators responded multiple times to the survey over the 5 years studied. This captures the yearly experience of the organization and the program administrator. Since the implementation start-up (prior to service) period can last from 6 to 12 months, the annual survey is a snapshot that provides a perspective of the administrators and the organizations as they move through the implementation stages and address the operational components to launch and sustain their programs.

Size, organizational structure, geographic region, and primary services provided (e.g., medical care, senior services, and housing repair) varied among these 65 organizations. Types of organizations implementing CAPABLE included the following: healthcare organizations (28–45%) (e.g., home healthcare agencies, or integrated hospital and clinic systems and rehabilitation, or long-term care facilities), housing service and home repair or construction organizations (15–39%) (e.g., Habitat for Humanity affiliate chapter), managed care organizations (3–6%) (e.g., Medicare Advantage health plan), community-based organization (22–28%) (e.g., Area Agencies on Aging and Meals on Wheels service provider), and government agencies and university research centers (6–9%). The size and type of organization did not appear to affect the ability to adopt CAPABLE. A key driver for adoption was initial funding, often from grants or private sources.

Of the 65 licensed organizations examined over 5 years, the stage of implementation and/or conclusion of their programs was as follows:

- 28 (43%) were licensed and were either preparing to or actively providing CAPABLE service, with 13 of these sites (20% of the licensed organizations) progressing from Stage 3 (initial implementation) to sustaining the program (Stage 4 or 5).
- 35 (54%) offered the program, came to the end of their license, and did not renew; that is, they ended after the implementation stage (Stage 3).
- 2 organizations (3%) were licensed—but never advanced and decided not to pursue CAPABLE.

One barrier faced by all of the sites operating during this 5-year period was the impact of COVID-19, which caused most of these organizations to pause their CAPABLE service for between 2 and 12 months from 2020 to 2021.

### Reach

As of 2024, a total of approximately 4,000 individuals completed CAPABLE. We calculated this total by adding up the reported number of participants who completed the CAPABLE service each year from each organization.

Based on funder and organizational criteria for participant selection set by the organizations studied, it is reasonable to assume that most of these participants were low-income (for example, parameters included criteria such as “less than 80% of the median income in the area”). Sites do not report demographic characteristics of their participants, so we do not have quantitative data on race, ethnicity, or language. However, qualitative information from the monthly calls indicates most (90% or more) of CAPABLE participants served spoke English as their primary language and most (75% or more) were White, with a few exceptions (one site served a majority of Black/African-American participants).

Service volume (number of completed CAPABLE participants) by site was generally between 10 and 40 individuals per year, but three sites provided service to between 80 and 100 people in 1 year and one site served 300 participants in a year.

Service volume was constrained by funding and staffing resources, as well as the funder prescribed parameters. Since the primary source of funding for most of these 65 organizations was grants, the grantor approved the grantee proposal around target number of people to be served within the defined timeframe. Typical volume targets were between 20 and 50 participants per year, with 1, 2, or 3 years of service funded. After that time, programs were expected to find other funding. However, during this timeframe, the pandemic impacted all programs. For example, in 2020, only eight sites (32% of the number of licensed sites at that time) had served 10 or more participants within the year, compared to the initial organizational targets of between 20 and 50 participants.

### Implementation

We examined the 12 implementation components probed in the annual survey. There was one program administrator designated for each organization. Twenty-five program administrators responded to the survey in 2020, 25 in 2021, 23 in 2022, and 28 in 2023. As mentioned, this includes multiple responses from some administrators over the 5 years as they continued the operations of their CAPABLE service. Some organizations responded only once as their site had just become licensed that year and were only beginning their second year at the time of this examination. The 2024 survey was not yet distributed for this examination.

Aggregating survey responses, we found consistency in several component ratings. Respondents most often rated “Getting Senior Leadership Support” and “Maintaining Fidelity to the CAPABLE protocol” as *Easy.* Respondents most often rated “Recruitment” and “Finding Sustainable Funding” as *Difficult*.

“Managing the Program” had mixed results in terms of level of effort and difficulty or ease. Program administrators explained in monthly calls and in open-text boxes on the survey that managing CAPABLE required more time than originally estimated to set up/launch this multi-component program.

Respondents identified “Recruitment and Obtaining Eligible Referrals” to CAPABLE as a common challenge. Building awareness within the external environment among referral sources required continual effort. Program administrators explained that their referral sources initially had difficulty understanding CAPABLE; referrals for CAPABLE needed to be fostered one person or referral source at a time. However, mature sites had overcome this hurdle and often had waiting lists. Referrals effectively came from a wide variety of sources, such as clinics, home health agencies, area agencies on aging, rehabilitation centers, care management agencies, home support service providers and housing and other community-based service providers, as well as word-of-mouth from past participants who had “graduated” from CAPABLE.

“Getting Technical Support” was rated as *Easy* and an important enabling factor to these organizations to implement CAPABLE effectively. Any licensed site can request technical support from the National CAPABLE Center or Johns Hopkins key technical support staff for CAPABLE at any time within the course of their license agreement. In addition, all organizations are provided with a comprehensive CAPABLE Implementation Manual, online training and manuals for the clinicians, and bi-monthly, monthly, or quarterly office hours for each of the four key roles in CAPABLE: Program Administrators, Occupational Therapists, Registered Nurses, and Handy workers/Construction Specialists. The CAPABLE National Center provided additional tools and resources as they were developed, such as *Evaluation Considerations, Fidelity Frequently Asked Questions,* the *Readiness Assessment*, and *Cost/Budget Guide*.

Tables 4, 5 provide more detail on 4 years of survey responses, by the implementation component probed.

**Table 4:** CAPABLE annual survey results—“easy” components—(are bolded in green).

Implementation components	2020	2021	2022	2023
% Responded “*very/somewhat easy*”				
Managing the program and overseeing the implementation of CAPABLE	**33%**	**50%**	**50%**	**47%**
**Getting senior leadership support**	***95%***	***81%***	***86%***	***80%***
Finding staff (OT, RN, handy worker)	***55%***	**30%**	**52%**	**34%**
Finding partners (e.g., healthcare and housing repair)	**64%**	**46%**	***73%***	**50%**
**Setting up the workflows/processes**	***67%***	***73%***	**54%**	***63%***
Recruiting participants and getting eligible referrals	**22%**	**28%**	**25%**	**26%**
**Securing legal, insurance, business, or HIPAA agreements**	***73%***	***69%***	***59%***	***60%***
Data—Setting up platform gathering and entering data	**41%**	**52%**	**44%**	***60%***
Evaluation—aggregating and evaluating the program	***59%***	**52%**	***60%***	**50%**
Participants completing the full program (at least 8 visits)	***82%***	**43%**	**47%**	**53%**
Developing a sustainable financial/funding model	**25%**	**23%**	**25%**	**27%**
**Ensuring fidelity; monitoring how well the CAPABLE model is followed**	***69%***	***76%***	***57%***	***63%***
**Obtaining implementation assistance and guidance from the team**	***89%***	*96%*	***88%***	***97%***

(1) the 2024 survey had not yet been administered at press time; (2) response indicator threshold the bold italic of 55% used to indicate a majority. and (3) Bolded text indicates highest ranking components over 4 years studied.

**Table 5:** CAPABLE annual survey results—“difficult” components—(are bolded in red).

Implementation components	2020	2021	2022	2023
% responded “*very/somewhat difficult*”				
Managing the program and overseeing the implementation of CAPABLE	***67%***	**50%**	**50%**	**47%**
Getting senior leadership support	**6%**	**14%**	**13%**	**13%**
**Finding staff (OT, RN, handy worker)**	**44%**	***70%***	**48%**	***64%***
Finding partners (e.g., healthcare and housing repair)	**34%**	***55%***	**27%**	**43%**
Setting up the workflows/processes	**33%**	**28%**	**47%**	**33%**
**Recruiting participants and getting eligible referrals**	***78%***	***72%***	***75%***	***66%***
Securing legal, insurance, business, or HIPAA agreements	**26%**	**31%**	**41%**	**33%**
Data—Setting up platform gathering and entering data	***59%***	**48%**	***56%***	**37%**
Evaluation—aggregating and evaluating the program	**41%**	**48%**	**40%**	**34%**
Participants completing the full program (at least 8 visits)	**19%**	***57%***	**53%**	**26%**
**Developing a sustainable financial/funding model**	***75%***	***77%***	***75%***	**54%**
Ensuring fidelity; monitoring how well the CAPABLE model is followed	**31%**	**24%**	**43%**	**30%**
Obtaining implementation assistance and guidance from the team	**12%**	**4%**	**12%**	**3%**

*Totals across both tables may not add to 100% as some respondents indicated “Do not Know”.

The annual survey also included questions on organizational readiness, internal factors, and external factors, corresponding to the 13 chosen CFIR constructs. We found consistency in positive responses around the following: the evidence/value of CAPABLE, external influences around aging in community, senior leadership support, knowledge, and champion acumen—all with a strong-to-moderate positive effect. We determined the direction of the effect by reviewing each organization’s responses to the implementation components (discussed earlier), the notes from “office hours” calls, and our knowledge of the organization over multiple years as we provided technical support and answered their questions. We found consistency in negative responses around the following: complexity of the program, internal infrastructure readiness that was lacking, difficulty executing (particularly with partners), and availability of resources (particularly staffing) with a moderate-to-strong negative effect (Table 6).

**Table 6:** Summary of CAPABLE organizational factors, by CFIR construct.

Construct	Synopsis of findings	Strength/direction—As determined through review of office hour notes, survey responses, and one-on-one technical assistance across the duration of the implementation experience
Evidence/value	Stakeholders perceived CAPABLE is a high-quality program with valid evidence. Administrators and senior leaders believe that the intervention will have desired outcomes.	*Strong positive effect* (all sites indicating positive factor, *N* = 65)
Complexity	A fairly substantial effort is required to begin CAPABLE. Complexity comes from the protocol/components (10 home visits in specific sequence by three professional disciplines), required training, and the administrative operational steps and resources required to build organizational infrastructure/capacity. Some of the perceived complexity emanates from organizational infrastructure that had to be built or modified before the program could start screening and serving participants. Elements that contributed to difficulty included: staffing, documentation/data tracking, and training.	*Moderate negative effect* (all implementing sites indicating complexity, *N* = 63)
Cost	Costs among the implementers serving 50 + people/year were about $3,800 to $4,500 per participant. Sites had budgeted for these costs, so this was a “neutral” factor for most sites, although the cost was high compared to other falls prevention evidence-based programming, affecting sustainability funding.	*Neutral* (most sites indicating cost within expected parameters, *N* = 45)
External influence	External influences included interest in “aging in community” by stakeholders and receptive funders, state and private foundation and grant initiatives, and policy directions that supported CAPABLE. National visibility/ exposure of the program and opportunity to access grant funds for training, evaluation, and initial implementation were all identified as influencing adoption. Potential for value-based payment was mentioned as motivating adoption of CAPABLE.	*Strong positive effect* (all sites indicating high value of the external influence factors, *N* = 65)
Organization	Organizational characteristics did not seem to influence the ability to adopt or launch CAPABLE.	*Neutral* (wide variety of organizations implemented CAPABLE with fidelity, *N* = 60)
Readiness	Respondents indicated a high degree of commitment around the decision to implement CAPABLE, however experience showed lack of readiness in key infrastructure or administrative areas, particularly management time, data/tracking systems, and marketing/outreach.	*Moderately negative effect* (survey shows self-reported readiness in the described areas as low in about two-thirds of sites, *N* = 50)
Senior leadership	Visible commitment and involvement of senior leaders and program managers was widely reported.	*Strong positive effect* (nearly all sites indicate senior leadership support and the importance of this support, *N* = 60)
Resources	Resources were sometimes less than needed; budgeting did not often cover administration-related expenses. Organizations had difficulty in finding clinical staff that were a good fit for the program’s person-directed approach. Organizations reported staff turnover challenges. Sustainability funding was a key challenge for most sites	*Moderately negative to strong negative effect* (nearly all sites indicate challenges with staffing at one point in their implementation, *N* = 60; Almost all sites indicated they did not have /could not identify sustainment funding *N* = 60)
Technical assistance	Reported ease of access to information, training, and technical support to support their CAPABLE	*Strong positive effect* (nearly all sites indicate high value of the ongoing technical support, *N* = 60)
Knowledge	Individuals placed a high value on the program. Key stakeholders had very strong positive attitudes toward CAPABLE.	*Positive effect* (Once the site administrators were oriented to the CAPABLE program, most indicated strong value on this knowledge, *N* = 55)
Planning	Organizations that mapped out an implementation plan with tasks and timeframe and workflows and that worked with the technical CAPABLE team were better prepared. Despite this, most sites expressed some challenges in organizational readiness or infrastructure to support CAPABLE processes and related data/tracking needs. Components mentioned as needing additional planning/development included: partnership development/business agreements, data collection/tracking/entry and information technology, evaluation, and assistance with marketing/outreach/referral/recruitment strategy.	*Mixed to moderately negative effect* (many sites indicating needing more planning and preparation time than anticipated, *N* = 60)
Champion	Every site appointed a person with responsibility for implementing CAPABLE. People serving as the administrator expressed enthusiasm for being able to implement CAPABLE. Some individuals reported having additional roles/responsibilities and they had to juggle time and focus with other duties.	*Positive effect* (many site administrators indicated high value of serving as a champion, *N* = 50)
Executing	Challenges to operationalize CAPABLE-often related to organizational capacity. External environmental issues, such as COVID, and internal organizational issues, such as changes in service strategy, budget or staffing, also affected execution.	*Mixed to negative effect* (nearly all sites indicated some challenges in executing, *N* = 55)

Program administrators saw themselves as champions with a strong perceived value of the CAPABLE program. They articulated how CAPABLE was a great fit with organizational strategy to promote “aging in community.” In calls, program managers indicated leadership support had been an important factor for adoption. Senior leaders, who were also occasionally involved in calls with the CAPABLE team, described the strategic importance and evidence base of CAPABLE—qualities that were emphasized early in the exploration and pre-adoption stages.

All CAPABLE program administrators described an operational learning curve to implement CAPABLE. CAPABLE requires a fairly substantial administrative effort to launch and manage this multi-component intervention, involving three professional disciplines and ten coordinated in-home visits over 4 months. Administrators described needing additional data tracking and evaluation infrastructure, staff resources, management time to oversee staff/contractors, planning support to create new policies or procedures to establish workflows, and help in monitoring the sequencing and scheduling of visits to ensure adherence to the protocol. Gaps in readiness around these infrastructure and resource components were not apparent to them prior to adopting the program.

Challenges with lack of infrastructure readiness to support the components of CAPABLE suggest some readiness factors may be under-appreciated. Self-reported organizational readiness was high in the pre-adoption phase. However, as the operational requirements of CAPABLE were better understood, program administrators discussed (in *ad hoc* and “office hours” monthly calls) areas where they realized their organizations lacked capacity.

### Effectiveness

Regarding effectiveness, all organizations use the same pre/post-measures of participant outcomes. In the annual survey, the organizational respondent affirms the measures they are using. These measures were consistent with the suggested measures to monitor participant outcomes pre- and post-service. These include the following:

- 8 ADL activities—level of difficulty from “No Difficulty” to “Unable to Do” using a 5-point scale.
- 8 IADL activities—level of difficulty from “No Difficulty” to “Unable to Do” using a 5-point scale,
- Depressive symptoms—using the PHQ-9 instrument, with the last item on suicide ideation not used (therefore, we refer to this as the PHQ-8 item set).
- Falls Efficacy—using the Falls Efficacy Scale—A 10 item scale by Mary Tinetti.

All administrators report on whether they have observed improvement in these participant outcomes, indicating in the aggregate whether they have seen overall positive changes, decline/negative changes, or no change. Where the organization conducted a more formal evaluation and shared that with the National Center (*N* = 15, unpublished), they reported the following results:

- Improvement in functional status (typically halving the functional status limitations reported comparing pre- and post-scores and aggregating across the participants served),
- Improvement in depression (reducing reported depressive symptoms by 20–40%), and
- Improving falls self-efficacy (improving confidence of not falling) by most of the CAPABLE participants served.

These results were similar to those demonstrated in the original CAPABLE studies and in subsequent studies of specific programs (12, 13, 15, 29).

We observed a gap in expertise around evaluation design and methods, data platforms, and ability to track and aggregate the pre- and post-outcome measure data. Several program administrators indicated these as areas of weakness, impacting their ability to evaluate results and demonstrate a return on investment of their particular program for potential payers. Potential payers in the United States would include managed care/health plans, particularly “Special Needs Plans” (SNPs) which are a type of Medicare Advantage plan that targets dually eligible individuals. The term dually eligible means a person is eligible for both Medicaid (state administered—for low-income people that meet the level of care needs and financial eligibility guidelines as set by the state) and Medicare (for all older adult citizens age 65 + and for younger people with physical disabilities who meet the federal guidelines for eligibility). It is a term widely used and understood in the United States but is not relevant to other countries. Other value-based payors in the U.S. would include Accountable Care Organizations (ACOs) which are usually integrated health delivery providers with an insurance component. Both SNPs and ACOs are unique to the United States healthcare delivery and payment system. They are designed to provide an incentive to better manage care by paying upfront for each enrolled individual, rather than based on fee-for-service utilization of that person. The idea is to promote prevention upstream, thereby avoiding higher cost events as disease or condition effects progress.

Regarding return on investment (ROI), CAPABLE has shown a 6- to 10-fold return on investment to the Medicare and Medicaid programs in other research, where a $3,000 (USD) investment resulted in almost $30,000 (USD) savings (14, 15). However, among these 65 sites, only a few had pursued calculating their own CAPABLE program return on investment—in part because it required following the participant for 24 months post-program graduation. The technical support team at the national program office has provided technical assistance on ROI calculation and encourages sites to determine the following:

- “Cost to whom”—Identify the stakeholder(s) of interest. In addition to the current funder/payer, sites are encouraged to identify costs that would be experienced by a potential payer, usually a managed care plan or state government. There are different cost scenarios and inputs based on each potential payer.
- “What costs”—Determine the input costs (other than direct costs of labor and supplies, if any) to be added to the equation.
- “What time period”—Determine the length of time to be used to calculate the cost and value.
- “Value”—Identify the benefits, calculate the value of these benefits, and identify to whom the benefits inure.

### Maintenance

Organizations report adherence to following the CAPABLE protocol with fidelity via an attestation at the end of the annual survey. All but two organizations attested to following the protocol with fidelity. The two organizations that were unable to follow the protocol had shortened the number of visits, due to resource constraints. Therefore, the participants were not considered to have received the full dose of CAPABLE, and the organizations’ licenses ended.

Regarding sustainability, administrators reported wanting to continue the service; however, sustainability was elusive for most. Among these 65 organizations, most received time-limited grant support to launch their CAPABLE programs. When the grant ended, they often needed to close their CAPABLE program (usually after 3 years). Despite extensive searching and additional grant requests or outreach to potential payers, they were not able to secure sustainment funding. Organizations explored value-based payments from Medicare Advantage plans, Accountable Care Organizations, or state agency grants or waiver programs to secure sustainable funding, but such funding was not forthcoming for most programs as of mid-2024.

Funding was the number one issue in maintenance, with two other key issues also consistently raised: maintaining the necessary staff; and recruitment of individuals who fit the criteria to participate in the program.

Based on the detailed data from various sources reported herein, we summarize results at the macro-level across all CAPABLE organizations using the five RE-AIM domains (Table 7).

**Table 7:** Summary of results by RE-AIM domain—evidence suggests.

Reach	Somewhat low penetration—initially difficult for sites to enroll sufficient number of people to reach their intended target (may take a year or so to establish referral base)
Effectiveness	High value of the program with demonstrated outcomes as expected for participants, as well as high degree of participant AND clinician satisfaction
Adoption	A wide variety of organizations can successfully adopt CAPABLE. Requires a fairly high level of organizational capacity, particularly if working with a new partner.
Implementation	Evidence that organizations are implementing all of the components of CAPABLE; there appears to be a high degree of adherence to the CAPABLE protocol
Maintenance	Long-term sustainability is challenging, primarily due to no consistent funding source.

## Discussion

This investigation identified key implementation factors supporting or hindering the delivery and sustainability of CAPABLE, demonstrated successful use of the two frameworks to guide the examination, and has offered an approach for ongoing implementation monitoring.

Senior leadership support was a strong driver of adoption. Leadership and funding commitment were critical to promote effective implementation and sustainability. Other studies have reported similar findings pertaining to senior leadership support (1, 3, 6, 27, 30). This underscores the importance of cultivating awareness and building internal support for the evidence-based program prior to launch, particularly in the C-Suite. Additional studies discuss similar readiness factors. For example, in a review of the implementation experience of the *Healthy Moves for Aging Well* program, a key challenge was engaging adoption by providers, and a key readiness factor was having senior leadership and program administrator support (31). In a comprehensive evaluation by the Centers for Medicare and Medicaid Services of 12 nationally disseminated programs, the evaluators found low existing market demand and program awareness (which affected recruitment) as well as a lack of a sustainable payment model to finance and support the delivery of these programs long term (32). A systematic review of evidence-based program sustainment found multiple facilitators (e.g., alignment) and barriers (funding, other limitations in resources) that affected ability to sustain the program (1, 33).

A program champion and effective program manager also were widely reported as facilitators of implementation success, consistent with other research (3, 34). Champions (program administrators) indicated their strong perceived value of the CAPABLE program. This suggests that to move from adoption to effective implementation, organizations should identify managers with a strong commitment to the program.

Findings around challenges with recruitment were consistent with other research and evaluation of evidence-based programs for older adults or caregivers (7, 31, 32). This suggests an important area for national focus—more resources are needed to achieve name recognition of CAPABLE and other evidence-based programs.

Most respondents needed additional infrastructure and resources compared to initial expectations. This is congruent with other studies probing characteristics and factors present among effective implementers and what readiness and supports are needed to facilitate effective implementation (6, 9, 31, 35). The organizational lift to build this program can be a restraining force in dissemination (36). Among these organizations, there was a frequently reported infrastructure readiness gap in evaluation design and data tracking capacity. A study of three states implementing evidence-based programs also found additional acumen around structured evaluation was needed (37).

A facilitating factor was technical support and training provided by Johns Hopkins and the CAPABLE National Center, as well as peer support through calls and/or learning collaboratives. Technical assistance and peer support have been shown to be important in other studies of implementation effectiveness (7).

### Potential impact

Given the strong and consistent participant outcomes observed in the CAPABLE organizations studied, we calculated the potential cost savings to Medicare and Medicaid if even just half of the 4,000 participants (2,000 individuals) were dually eligible and had achieved the mean cost savings identified in previous studies (approximately $30,000 USD for both Medicare and Medicaid). This would represent a cost savings of $60 million USD, in 2017 dollars. These savings are driven by better management of function at home, which avoids expensive falls and other healthcare utilization in the 24 months following the intervention. Notably, this figure does not calculate additional value to the community (for example, the value of avoiding persistent emergency medical and fire department calls when the person who lives alone falls). Moreover, there are important benefits to the participants that should be considered, such as the ability to stay in their residences, avoidance of pain and suffering associated with falls and other injuries, and emotional and health benefits of the positive behavior change aspects of CAPABLE, which are considered the essence of the program driving improved self-care. As of June 2024, there were 12.8 million dually eligible (for Medicare and Medicaid) individuals in the U.S (38). If even one-quarter (3.2 million individuals) could access a CAPABLE program, the estimated cost savings to Medicare and Medicaid would be $96 billion USD.

Despite the strong evidence of substantial positive health outcomes and of potential cost savings to federal and state governments for older adults participating in CAPABLE, policy and payment to ensure sustainment funding has not materialized for most programs. The organizations bearing the cost of CAPABLE do not receive the benefits of costs avoided. This is regrettable, especially given the laudable effort to stand up a CAPABLE program and achieve the effect in health outcomes as were demonstrated in the initial research studies. Over and over, organizations small and large, across all regions, are effectively implementing CAPABLE in their initial implementation (Stage 3) and older adults in their communities who participate describe transformational improvement in their lives. When grant funding ends, however, the organization finds it has to dismantle the program given lack of funding. This is a barrier most acutely observed in the United States, where healthcare policy and payment does not support upstream action focused on improving functional ability in people with chronic conditions. This has been described as a “wrong pocket” problem (39). Notably, the one program implemented in Nova Scotia, Canada, has been successfully implemented and is being expanded.

### Framework utility

The combination of RE-AIM and CFIR for studying implementation of CAPABLE offered great utility. RE-AIM provided a framework for looking at results. CFIR guided examination of context. The two frameworks helped identify factors important in readiness and effective implementation, factors also found to be important in other studies (19, 24, 31). Others using both frameworks have also identified RE-AIM as being useful for evaluating change to assure external validity, while CFIR helps explain *why* implementation succeeded or failed (27).

To replicate the methods used, a research team would choose the indicators that are relevant for each of the RE-AIM domains and identify data sources that capture qualitative and quantitative information on those indicators. The research team would also identify key implementation components or actions required to implement the program, and the internal and external factors that are relevant and observed in implementing the program. The research team would then embed the components and constructs into an annual survey that would be completed by the lead administrator or operational manager at each program site. Each site would respond to the survey every year that it operated the program, allowing for identification of trends and patterns over time. There is utility in continuing to ask administrators annually about their implementation experience as ease or difficulty with different components varies from year to year. This provides important information when taking a longitudinal and ongoing approach to studying implementation and is recommended to others as a strategy. Ideally, the research team would provide ongoing technical assistance and facilitate regular shared learning “office hours” calls with all implementation sites to capture contextual information in real time. This unstructured feedback provides many insights into challenges and strategies as they emerge.

The CAPABLE National Center will continue to use the two frameworks to take a structured and consistent look at implementation experience. Others have recommended that organizations implementing evidence-based programs regularly gather and report metrics on implementation experience and program outcomes, guided by theories, models, and frameworks (40). We recommend that evidence-based program stewards use frameworks such as RE-AIM and CFIR to set up a structured reporting system that gathers information not only on participant outcomes but also on organizational implementation experience such as demonstrated in this example. Structured annual reporting coupled with capture of qualitative information provides an important feedback loop to the program steward, allows for benchmarking by and for the implementing organizations, and can guide informed decision-making on where adaptations or other changes may be needed. We would highly recommend this as a strategy. Other national program offices are encouraged to conduct these virtual meetings/calls of all implementation sites to facilitate effective implementation and promote ongoing shared learning around a specific evidence-based program such as CAPABLE. This offers real-time insight and an ongoing opportunity to identify barriers and facilitators of progress and address needs proactively through enhanced technical support.

### Strengths and limitations

Strengths include the embedded research approach and use of multiple sources to capture annual implementation experience. Relatedly, identifying key implementation components and important constructs based on first-hand knowledge of organizational experience is a strength. Probing implementation experience and constructs annually through structured data collection from all sites provided consistent snapshots that could be aggregated. Collecting information from the key operational lead within each organization using forced choice response scales and open-ended response provided information from those most directly responsible for the implementation process. In addition, strengths of the research team included having ongoing technical knowledge from working day-to-day with the program sites and extensive experience with the intervention over many years. The primary author (DP) is an embedded researcher and provides day-to-day technical assistance to CAPABLE implementation organizations. The other authors either co-developed the CAPABLE program (SS, LG), have worked with CAPABLE sites around a pilot study or to support a Learning Collaborative (JS), or have evaluated evidence-based programs nationwide using implementation frameworks (MS).

Limitations include our inability to reach five organizations that had ended their programs and the few non-responders (less than 10% per year) to our annual survey. We do not know whether their experience would be substantively different from the organizations included in our analysis.

## Conclusion

We examined factors that advanced or impeded implementation and sustainability of the evidence-based program CAPABLE by 65 organizations over 5 years, using the RE-AIM and CFIR frameworks to guide our examination. Through an annual e-survey of all licensed organizations and review of notes from monthly office hours and *ad hoc* calls, we conducted a structured examination performing qualitative thematic and descriptive analysis. Factors consistently supporting implementation included senior leadership support, technical assistance, and the protocol of the program, which guided fidelity adherence. Conversely, common challenges included difficulty with recruitment and hiring/finding the required personnel. Internal factors supporting readiness and adoption were perceived value of the program and program manager knowledge and commitment. External factors supporting adoption were initial funding to start a pilot and strategic tie to aging in community organizational commitments.

Lack of sustainability funding was the greatest factor impacting sustainment. This is an ongoing challenge. There are some encouraging signs as several government agencies and policymakers in multiple states in the U.S. are working on ways to incorporate CAPABLE into a list of approved benefits under certain circumstances (for example, via waiver program under the state’s Medicaid funding for low-income older adults). Raising awareness of the program to make it easier to reach potential participants who then agree to enroll in CAPABLE is another factor that would enhance sustainability.

Based on the learning acquired and ongoing insights from monthly technical assistance calls, the CAPABLE National Center is adding to the readiness guidance for organizations adopting CAPABLE to promote ease of implementation and to build the organizations’ acumen for conducting evaluations that supply potential payers with cost and benefit calculations as well as evidence on participant outcomes achieved.

This study provides a use case for employing the RE-AIM and CFIR frameworks to track ongoing implementation. We offer practical ways to monitor, evaluate, and report on ongoing implementation of evidence-based programs.

## Data Availability

The datasets presented in this article are not readily available because licensure data is restricted. Requests to access the datasets should be directed to dpaone1@jhu.edu.

## Footnotes

^1^ www.re-aim.org

^2^ www.cfirguide.org
